# Hydrogen-bond inter­actions in morpholinium bromide

**DOI:** 10.1107/S1600536811035598

**Published:** 2011-09-14

**Authors:** Kamentheren Padayachy, Zolani Mgcima, Manuel A. Fernandes, Helder M. Marques, Alvaro S. de Sousa

**Affiliations:** a School of Chemistry, Molecular Sciences Institute, University of the Witwatersrand, Private Bag 3, Wits 2050, Johannesburg, South Africa

## Abstract

In the title compound, C_4_H_10_NO^+^·Br^−^, which was synthesized by dehydration of diethano­lamine with HBr, morpholinium and bromide ions are linked into chains by N—H⋯Br hydrogen bonds describing a *C*_2_^1^(4) graph-set motif. Weaker bifurcated N—H⋯Br inter­actions join centrosymmetrically related chains through alternating binary graph-set *R*_4_^2^(8) and *R*_2_^2^(4) motifs, to form ladders along [100]. In addition, C—H⋯O inter­actions between centrosymmetric morpholinium cations link ladders, *via* 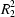
(8) motifs, to yield sheets parallel to (101), which in turn are crosslinked by weak C—H⋯O inter­actions, related across a glide plane, to form a three-dimensional network.

## Related literature

For the structures of related morpholinium salts, see: Loehlin & Okasako (2007 ▶); Mafud *et al.* (2011 ▶); Swaminathan *et al.* (1976 ▶); Koroniak *et al.* (2000 ▶); Turnbull (1997 ▶); Mazur *et al.* (2007 ▶); Yao (2010 ▶); Christensen *et al.* (1993 ▶). For the synthesis, see: Pettit *et al.* (1964 ▶). For the graph-set analysis, see: Bernstein *et al.* (1995 ▶).
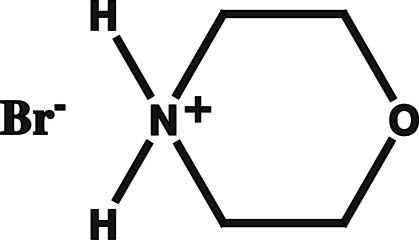


## Experimental

### 

#### Crystal data

C_4_H_10_NO^+^·Br^−^
*M* *_r_* = 168.04
Monoclinic, 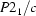

*a* = 6.1247 (2) Å
*b* = 10.3063 (3) Å
*c* = 10.1141 (3) Å
β = 100.312 (2)°
*V* = 628.12 (3) Å^3^
*Z* = 4
Mo *K*α radiation
μ = 6.44 mm^−1^
*T* = 173 K
0.40 × 0.20 × 0.09 mm

#### Data collection

Bruker APEXII CCD area-detector diffractometer
Absorption correction: integration (face indexed absorption corrections carried out with *XPREP*; Sheldrick, 2008 ▶) *T*_min_ = 0.183, *T*_max_ = 0.595
11662 measured reflections
1516 independent reflections
1314 reflections with *I* > 2σ(*I*)
*R*_int_ = 0.210

#### Refinement

*R*[*F* ^2^ > 2σ(*F* ^2^)] = 0.039
*wR*(*F* ^2^) = 0.100
*S* = 1.03
1516 reflections
64 parameters
H-atom parameters constrained
Δρ_max_ = 1.07 e Å^−3^
Δρ_min_ = −1.38 e Å^−3^

### 

Data collection: *APEX2* (Bruker, 2005 ▶); cell refinement: *APEX2*; data reduction: *SAINT-Plus* (Bruker, 2005 ▶); program(s) used to solve structure: *SHELXS97* (Sheldrick, 2008 ▶); program(s) used to refine structure: *SHELXL97* (Sheldrick, 2008 ▶) and *WinGX* (Farrugia, 1997 ▶); molecular graphics: *SHELXTL* (Sheldrick, 2008 ▶) and *PLATON* (Spek, 2009 ▶); software used to prepare material for publication: *SHELXTL*.

## Supplementary Material

Crystal structure: contains datablock(s) global, I. DOI: 10.1107/S1600536811035598/lr2027sup1.cif

Structure factors: contains datablock(s) I. DOI: 10.1107/S1600536811035598/lr2027Isup2.hkl

Supplementary material file. DOI: 10.1107/S1600536811035598/lr2027Isup3.cml

Additional supplementary materials:  crystallographic information; 3D view; checkCIF report

## Figures and Tables

**Table 1:** Hydrogen-bond geometry (Å, °)

*D*—H⋯*A*	*D*—H	H⋯*A*	*D*⋯*A*	*D*—H⋯*A*
N1—H1*A*⋯Br1	0.92	2.52	3.331 (2)	148
N1—H1*A*⋯Br1^i^	0.92	2.89	3.389 (2)	115
N1—H1*B*⋯Br1^ii^	0.92	2.40	3.292 (2)	164
C4—H4*A*⋯O1^iii^	0.99	2.52	3.366 (4)	143
C1—H1*C*⋯O1^iv^	0.99	2.59	3.498 (4)	152

Symmetry codes: (i) 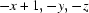; (ii) 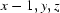; (iii) 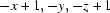; (iv) 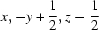.

**Fig. 1.**
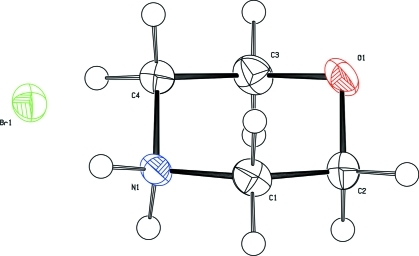
Molecular structure of (1), showing the atomic numbering scheme. Displacement ellipsoids are drawn at the 50% probability level.

**Fig. 2.**
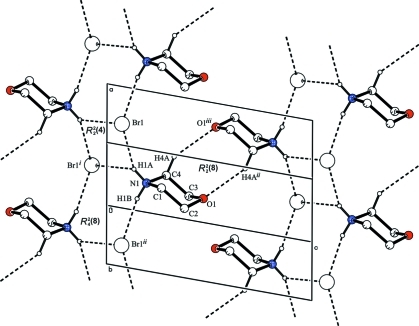
Ring motifs (a) R22(4) and (b) R42(8) arising from intermolecular N-H···Br, and (c) R22(8) from C-H···O interactions, in sheets parallel to the ac plane. [symmetry codes: (i) 1-x,-y,-z; (ii) x-1,y,z; (iii) 1-x, -y,1-z;]

**Fig. 3.**
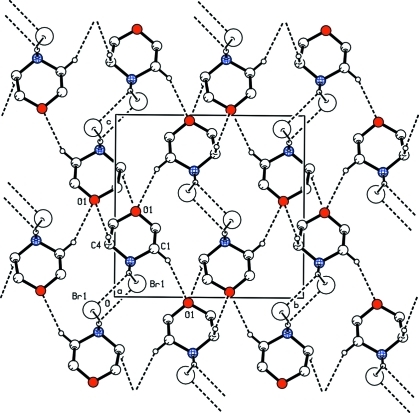
Intermolecular C1-H1C···O1 interactions linking sheets into a three dimensional network.

## supplementary crystallographic information

### Comment

The structures and hydrogen bonding of several salts of morpholinium and its derivatives have been reported (Loehlin & Okasako, 2007; Mafud *et al.*, 2011; Swaminathan *et al.*, 1976; Koroniak *et al.*, 2000 ; Turnbull, 1997; Mazur *et al.*, 2007; Yao, 2010; Christensen *et al.*, 1993). The title compound, morpholinium bromide, contains a single quaternary nitrogen donor (Figure1) and weak N-H···Br interactions are observable in the crystal structure. The morpholinium and bromide ions are joined into chains along the *a*-axis through N-H···Br hydrogen bonds in a motif of graph set *C*_2_^1^(4). Chains are joined to form ladders by weak, bifurcated N-H···Br interactions at ammonium hydrogen, H1A, (Figure 2). Alternating ring motifs *R*_4_^2^(8) and *R*_2_^2^(4) describe the binary graph-set for ladders along [100]. Weak C4-H4A···O1 interactions between centrosymmetric morpholinium cations link ladders,*via R*_2_^2^(8) motifs, to yield sheets parallel to the *ac*  plane, which in turn are weakly joined by C1-H1C···O1 interactions (Figure 3) across a glide plane perpendicular to [010], glide component (0, 0, 0.5), to form a three dimensional network.

### Experimental

Morpholinium Bromide was obtained as the minor product of the synthesis reported by Pettit *et al.* (1964). Diethanolamine (12 g, 0.114 mol) was added, with cooling, to 100 mL of 48% HBr. The reaction vessel was fitted with a Vigreaux column and Dean-Stark apparatus and the solution heated collecting approximately 70 mL of water through azeotropic distillation. The remaining HBr was removed under reduced pressure to yield viscous orange oil that crystallized upon cooling. The pure crystalline sample product was obtained by several recrystallisations from an ethanol-diethyl ether solution.

^1^H(D_2_O, 300 MHz) 3.667 (4*H*, t, CH_2_NH), 3.779 (4*H*, t, CH_2_O).

### Refinement

Hydrogen atoms were visible in the difference map and those bonded to carbon atoms were positioned geometrically and allowed for as riding atoms with C—H = 0.99 Å (CH_2_) and N—H = 0.92 Å (NH_2_). The coordinates of hydrogen atoms involved in hydrogen bonding were refined freely. During the refinements the *U_iso_*(H) values were set at 1.2*U_eq_* of the parent atom.

### Crystal data

**Table:**

C_4_H_10_NO^+^·Br^−^	*F*(000) = 336
*M**_r_* = 168.04	*D*_x_ = 1.777 Mg m^−^^3^
Monoclinic, *P*2_1_/*c*	Mo *K*α radiation, λ = 0.71073 Å
Hall symbol: -P 2ybc	Cell parameters from 5677 reflections
*a* = 6.1247 (2) Å	θ = 2.9–28.3°
*b* = 10.3063 (3) Å	µ = 6.44 mm^−^^1^
*c* = 10.1141 (3) Å	*T* = 173 K
β = 100.312 (2)°	Needle, colourless
*V* = 628.12 (3) Å^3^	0.40 × 0.20 × 0.09 mm
*Z* = 4	

### Data collection

**Table:**

Bruker APEXII CCD area-detector diffractometer	1516 independent reflections
Radiation source: fine-focus sealed tube	1314 reflections with *I* > 2σ(*I*)
graphite	*R*_int_ = 0.210
φ and ω scans	θ_max_ = 28.0°, θ_min_ = 2.9°
Absorption correction: integration (face indexed absorption corrections carried out with *XPREP*; Sheldrick, 2008)	*h* = −8→8
*T*_min_ = 0.183, *T*_max_ = 0.595	*k* = −13→13
11662 measured reflections	*l* = −13→13

### Refinement

**Table:**

Refinement on *F*^2^	Primary atom site location: structure-invariant direct methods
Least-squares matrix: full	Secondary atom site location: difference Fourier map
*R*[*F*^2^ > 2σ(*F*^2^)] = 0.039	Hydrogen site location: inferred from neighbouring sites
*wR*(*F*^2^) = 0.100	H-atom parameters constrained
*S* = 1.03	*w* = 1/[σ^2^(*F*_o_^2^) + (0.0518*P*)^2^] where *P* = (*F*_o_^2^ + 2*F*_c_^2^)/3
1516 reflections	(Δ/σ)_max_ = 0.002
64 parameters	Δρ_max_ = 1.07 e Å^−^^3^
0 restraints	Δρ_min_ = −1.38 e Å^−^^3^

### Special details

**Table:**

Experimental. Intensity data were collected on a Bruker APEX II CCD area detector diffractometer with graphite monochromated Mo Kα radiation (50 kV, 30 mA) using the APEX 2 (Bruker, 2005) data collection software. The collection method involved ω-scans of width 0.5° and 512 x 512 bit data frames. Data reduction was carried out using the program SAINT-Plus (Bruker, 2005). The crystal structure was solved by direct methods using SHELXTL (Sheldrick, 2008). Non-hydrogen atoms were first refined isotropically followed by anisotropic refinement by full matrix least-squares calculations based on F^2^ using SHELXTL.
Geometry. All esds (except the esd in the dihedral angle between two l.s. planes) are estimated using the full covariance matrix. The cell esds are taken into account individually in the estimation of esds in distances, angles and torsion angles; correlations between esds in cell parameters are only used when they are defined by crystal symmetry. An approximate (isotropic) treatment of cell esds is used for estimating esds involving l.s. planes.
Refinement. Refinement of F^2^ against ALL reflections. The weighted R-factor wR and goodness of fit S are based on F^2^, conventional R-factors R are based on F, with F set to zero for negative F^2^. The threshold expression of F^2^ > 2sigma(F^2^) is used only for calculating R-factors(gt) etc. and is not relevant to the choice of reflections for refinement. R-factors based on F^2^ are statistically about twice as large as those based on F, and R- factors based on ALL data will be even larger.

### Fractional atomic coordinates and isotropic or equivalent isotropic displacement parameters (Å^2^)

**Table:**

	*x*	*y*	*z*	*U*_iso_*/*U*_eq_	
Br1	0.74904 (4)	0.11971 (3)	0.07284 (3)	0.02454 (16)	
O1	0.2489 (4)	0.10940 (17)	0.4694 (3)	0.0263 (5)	
N1	0.2813 (4)	0.0830 (2)	0.1937 (2)	0.0199 (5)	
H1A	0.3739	0.0758	0.1318	0.024*	
H1B	0.1372	0.0808	0.1480	0.024*	
C1	0.3228 (5)	0.2082 (3)	0.2657 (3)	0.0243 (6)	
H1C	0.2788	0.2807	0.2024	0.029*	
H1D	0.4831	0.2170	0.3026	0.029*	
C4	0.3199 (5)	−0.0280 (3)	0.2893 (3)	0.0246 (6)	
H4A	0.4801	−0.0347	0.3272	0.029*	
H4B	0.2732	−0.1098	0.2410	0.029*	
C2	0.1926 (5)	0.2149 (3)	0.3781 (3)	0.0273 (6)	
H2A	0.2241	0.2980	0.4268	0.033*	
H2B	0.0319	0.2119	0.3404	0.033*	
C3	0.1915 (5)	−0.0094 (3)	0.4004 (3)	0.0272 (6)	
H3A	0.0308	−0.0095	0.3628	0.033*	
H3B	0.2221	−0.0824	0.4646	0.033*	

### Atomic displacement parameters (Å^2^)

**Table:**

	*U*^11^	*U*^22^	*U*^33^	*U*^12^	*U*^13^	*U*^23^
Br1	0.0153 (2)	0.0332 (2)	0.0242 (2)	−0.00109 (9)	0.00118 (14)	−0.00764 (10)
O1	0.0358 (13)	0.0301 (11)	0.0127 (10)	0.0010 (8)	0.0031 (9)	0.0004 (7)
N1	0.0159 (11)	0.0289 (11)	0.0147 (11)	0.0018 (8)	0.0021 (9)	−0.0013 (8)
C1	0.0259 (14)	0.0220 (13)	0.0238 (14)	−0.0055 (10)	0.0014 (11)	0.0010 (10)
C4	0.0274 (14)	0.0238 (14)	0.0213 (14)	0.0042 (10)	0.0014 (11)	−0.0004 (10)
C2	0.0336 (16)	0.0246 (14)	0.0225 (15)	0.0050 (11)	0.0018 (13)	−0.0047 (11)
C3	0.0340 (15)	0.0245 (14)	0.0230 (14)	−0.0033 (11)	0.0053 (12)	0.0037 (11)

### Geometric parameters (Å, °)

**Table:**

O1—C3	1.422 (3)	C1—H1D	0.9900
O1—C2	1.428 (3)	C4—C3	1.495 (4)
N1—C1	1.481 (3)	C4—H4A	0.9900
N1—C4	1.490 (3)	C4—H4B	0.9900
N1—H1A	0.9200	C2—H2A	0.9900
N1—H1B	0.9200	C2—H2B	0.9900
C1—C2	1.502 (4)	C3—H3A	0.9900
C1—H1C	0.9900	C3—H3B	0.9900
			
C3—O1—C2	109.1 (2)	N1—C4—H4B	109.6
C1—N1—C4	110.9 (2)	C3—C4—H4B	109.6
C1—N1—H1A	109.5	H4A—C4—H4B	108.1
C4—N1—H1A	109.5	O1—C2—C1	110.8 (2)
C1—N1—H1B	109.5	O1—C2—H2A	109.5
C4—N1—H1B	109.5	C1—C2—H2A	109.5
H1A—N1—H1B	108.1	O1—C2—H2B	109.5
N1—C1—C2	110.1 (2)	C1—C2—H2B	109.5
N1—C1—H1C	109.6	H2A—C2—H2B	108.1
C2—C1—H1C	109.6	O1—C3—C4	111.3 (2)
N1—C1—H1D	109.6	O1—C3—H3A	109.4
C2—C1—H1D	109.6	C4—C3—H3A	109.4
H1C—C1—H1D	108.1	O1—C3—H3B	109.4
N1—C4—C3	110.2 (2)	C4—C3—H3B	109.4
N1—C4—H4A	109.6	H3A—C3—H3B	108.0
C3—C4—H4A	109.6		
			
C4—N1—C1—C2	−52.4 (3)	N1—C1—C2—O1	57.9 (3)
C1—N1—C4—C3	52.1 (3)	C2—O1—C3—C4	62.3 (3)
C3—O1—C2—C1	−62.4 (3)	N1—C4—C3—O1	−57.4 (3)

### Hydrogen-bond geometry (Å, °)

**Table:**

*D*—H···*A*	*D*—H	H···*A*	*D*···*A*	*D*—H···*A*
N1—H1A···Br1	0.92	2.52	3.331 (2)	148
N1—H1A···Br1^i^	0.92	2.89	3.389 (2)	115
N1—H1B···Br1^ii^	0.92	2.40	3.292 (2)	164
C4—H4A···O1^iii^	0.99	2.52	3.366 (4)	143
C1—H1C···O1^iv^	0.99	2.59	3.498 (4)	152

Symmetry codes: (i) −*x*+1, −*y*, −*z*; (ii) *x*−1, *y*, *z*; (iii) −*x*+1, −*y*, −*z*+1; (iv) *x*, −*y*+1/2, *z*−1/2.
